# Inhibition of A20 expression in tumor microenvironment exerts anti-tumor effect through inducing myeloid-derived suppressor cells apoptosis

**DOI:** 10.1038/srep16437

**Published:** 2015-11-12

**Authors:** Bin Shao, Xiawei Wei, Min Luo, Jiayun Yu, Aiping Tong, Xuelei Ma, Tinghong Ye, Hongxin Deng, Yaxiong Sang, Xiao Liang, Yu Ma, Qinjie Wu, Wei Du, Jing Du, Xiang Gao, Yi Wen, Ping Fu, Huashan Shi, Shuntao Luo, Yuquan Wei

**Affiliations:** 1 Division of Nephrology of Department of Internal Medicine and Lab of Aging Research, State Key Laboratory of Biotherapy & Collaborative Innovation Center of Biotherapy, West China Hospital, Sichuan University, Chengdu, Sichuan 610041, PR China; 2 College of life science, Sichuan University, Chengdu 610041, China

## Abstract

Myeloid-derived suppressor cells (MDSCs) are known to play important roles in the development of immunosuppressive tumor microenvironment. A20 is a zinc-finger protein which could negatively regulate apoptosis in several cell types. However, the role of A20 in tumor microenvironment remains largely unknown. In this study, we found that A20 was over-expressed in MDSCs. The treatment of tumor-bearing mice with small interfering RNA targeting A20 (si-A20) inhibited the growth of tumors. The infiltration of MDSCs was dramatically reduced after si-A20 treatment, as compared to control groups, whereas the numbers of dendritic cells and macrophages were not affected. Also, injection of si-A20 improved T cell mediated tumor-specific immune response. Depletion of MDSCs with anti-Gr1 antibody showed similar antitumor effect and improved T cell response. TNF-α was highly expressed after si-A20 injection. Furthermore, si-A20 induced apoptosis of MDSCs in the presence of TNF-α both *in vivo* and *in vitro*. Cleaved Caspase-3 and Caspase-8 were elevated with the activation of JNK pathway after the induction of MDSC apoptosis by si-A20. Thus, our findings suggested that knockdown of A20 in tumor site inhibited tumor growth at least through inducing the apoptosis of MDSCs. A20 might be a potential target in anticancer therapy.

Tumor microenvironment is an indispensable participant in the neoplastic process, fostering proliferation, survival and migration^1^. Tumor microenvironment contains many distinct types of cells, including endothelial cells and their precursors, fibroblasts and immune cells^1^,^2^. The malignant properties of cancer cells cannot be manifested without the important interplay with their local microenvironment^3^. Infiltration of different types of host cells in tumors leads to an evolving stromal compartment intermingled with tumor cells. This co-evolution of tumor cells and their microenvironment creates a permissive environment for the invasion of (epi) genetically altered tumor cells and a suppressive environment for the infiltrated host cells^4^,^5^. In this suppressive environment, infiltrated host cells are enslaved and even mutated by tumor cells to promote their growth and invasion, which make them different from normal host cells^6^,^7^. Thus, tumor microenvironment is so important to tumor development that any changes made to alter tumor microenvironment could be the approach to counteract tumor growth.

MDSCs play important roles in tumor microenvironment. In mice, MDSCs are characterized by the co-expression of the myeloid lineage differentiation antigen Gr1 and CD11b^8^. They suppress the anti-tumor immunity of T cells by inhibiting the activation and proliferation of CD4^+^ and CD8^+^ T cells^9^,^10^ and inducing regulatory T cells (Tregs)^11^. In addition, MDSCs also inhibit the cytotoxicity of natural killer-cell (NK) against tumor cells and block the production of IFN-γ^12^,^13^. Thus, the immunosuppressive functions of MDSCs make them main barriers in achieving efficient immunotherapies, such as cancer vaccines, where immunocompetent host is required to enhance the efficiency of the vaccines^14^. Thus, development of therapeutic strategy to target MDSCs has drawn considerable interest in the field of cancer therapy.

A20 (also known as TNFAIP3) is originally identified as a primary TNF-α responsive gene in human umbilical vein endothelial cells (HUVEC)^15^. A20 gene encodes a 790-amino acid zinc finger protein^16^, which negatively regulates inflammation, innate immunity and adaptive immunity^17^,^18^. A20 has also been well established as a negative feedback loop to inhibit NF-κB activation in response to tumor necrosis factor and several other stimuli^19^,^20^. Mice deficient for A20 are hyper-sensitive to TNF and die shortly after birth for severe inflammation and tissue damage in multiple organs, which suggests the potent anti-inflammatory function of A20^21^. In addition, recent evidence implicated that single nucleotide polymorphisms (SNPs) at the A20 locus are associated with some autoimmune diseases, including rheumatoid arthritis^22^ and systemic lupus erythematosus^23^. A20 has also been proposed to be related to several types of cancers^24^,^25^,^26^. Furthermore, the role of A20 in tumorigenesis might be cell type-dependent. However, how A20 expression in tumor microenvironment contributes to the pathophysiology of tumor remains largely unknown.

Due to the immunosuppressive characteristic of MDSCs, it is conceivable that MDSCs might express A20 in the tumor microenvironment and the knockdown of A20 might contribute to the elimination of MDSCs, which would hopefully improve anti-tumor immune response and exert anti-tumor effect. To test this concept, we detected whether or not the infiltrated immune cells in tumors were A20 positive. In this study, we especially focused on the role of A20 in the tumor microenvironment. To exclude the effect of A20 on tumor cells in this study, A20 negative tumor cell lines (E.G7, B16-F10) were selected and used. We treated E.G7 bearing mice with si-A20 and observed how it affected MDSCs and tumor microenvironment.

## Results

### A20 is highly expressed in tumor microenvironment and the knockdown of A20 eliminates MDSCs

**Figure 1:**
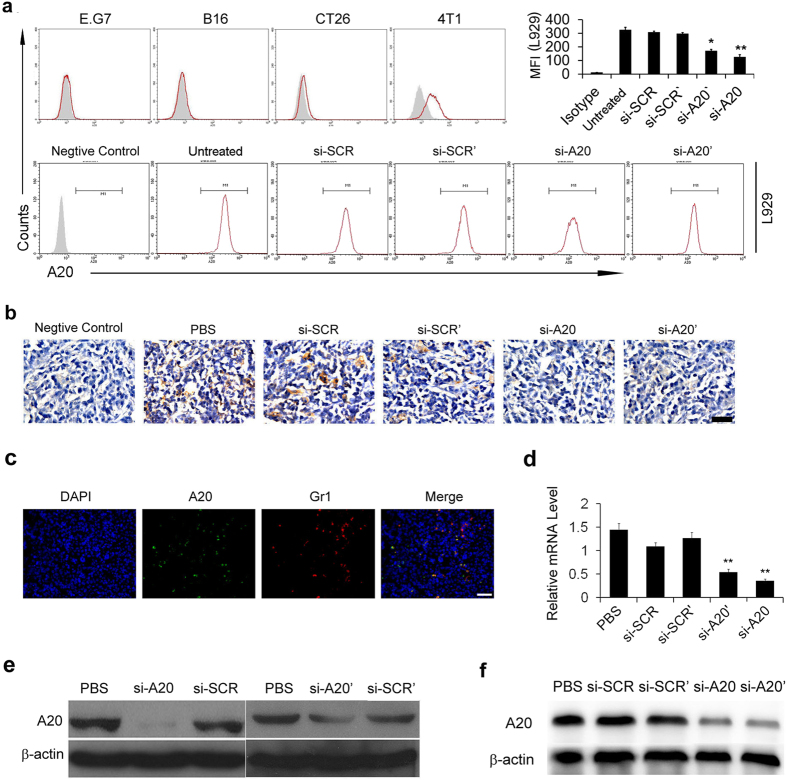
A20 expression in tumor microenvironment and the knockdown of A20 by si-RNA. (**a**) The expression of A20 in different cell lines. A20 expression was tested by flow cytometry in E.G7, B16-F10, CT26, 4T1 and L929 cell lines. Total number of 30000 cells was analyzed. The grey area represents the isotype control. si-A20 was used to test the specificity of the staining. The efficiency of knockdown of A20 was illustrated as reduction in mean fluorescence intensity. (**b**) Representative IHC analysis of A20 in E.G7 tumor sections (*n* = 5). Scale bar 50 μm. (**c**) The immunofluorescence staining of A20-positive and Gr1-positive cells. E.G7 tumor frozen sections were stained with DAPI (blue), Gr1 (Alexa-595) and A20 (FITC) (*n* = 5). Scale bar 50μm. (**d,e**) Knockdown of A20 by si-RNA in L929 cell lines was confirmed by qRT-PCR and western blotting. L929 cells were transfected with si-RNA *in vitro* and the mRNA level and protein level of A20 were detected. (**f**) Decrease of A20 expression in tumor tissue after si-RNA treatment. E.G7 tumor-bearing mice were treated with si-RNA intratumorally and CD11b^+^ cells were isolated from pooled tumor tissue suspension (n = 3–5). Data were obtained from two independent experiments. Data represent means ± SD. *p < 0.05 compared to si-SCR’ group, **p < 0.01 compared to si-SCR (ANOVA test).

**Figure 2:**
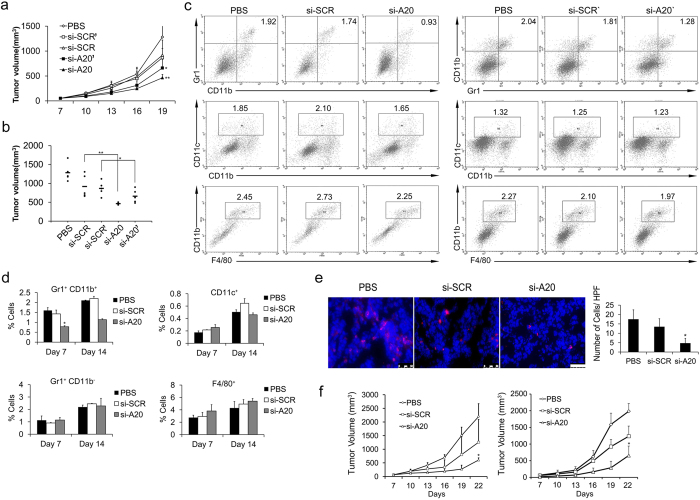
Si-A20 treatment in mice inhibits tumor growth and reduces tumor-associated MDSCs. (**a**,**b**) Treatment of mice with si-RNA inhibited E.G7 tumor growth (n = 5). Tumor volume of each mouse was valued on Day 19. (**b**) Data are representative of two independent experiments (n = 5). Data represent means ± SD. *p < 0.05 compared to si-SCR’, **p < 0.01 compared to si-SCR (ANOVA test). (**c**) Analysis of infiltrated immune cells in tumor after si-RNA treatment. Tumors were subjected to flow cytometry assay after si-RNA treatment on Day 14. Total number of 30000 cells was analyzed. Numbers illustrated indicate the percentage of the cells in total cells. Data are representative of two independent experiments (n = 3). (**d**) The proportions of Gr1^+^CD11b^+^ MDSCs, Gr1^+^CD11b^−^ granulocytes, CD11c^+^ DCs, F4/80^+^ macrophages were analyzed by flow cytometry on Day 7 and 14 after treatment (n = 3). (**e**) The reduction of MDSCs confirmed by immunofluorescence staining in tumor section. The positive cells in ten high powered fields (HPF) were counted (n = 5). Scale bar 50 μm. (**f**) Si-A20 treatment inhibited E.G7 (left) and B16-F10 (right) tumor growth in mice (n = 5). Data are representative of two independent experiments. Data represent means ± SD. *p < 0.05 compared to si-SCR (ANOVA test).

In order to study the role of A20 in the tumor microenvironment, several mouse tumor cell lines were used, including E.G7 (lymphoma), B16-F10 (melanoma), CT26 (colon cancer) and 4T1 (breast cancer). We first selected the A20 negative tumor cell lines to exclude the effect of A20 on tumor cells for *in vivo* study. We examined the expression of A20 protein in E.G7, B16-F10, CT26, 4T1 cells and the results showed that E.G7 and B16-F10 cells were detected negative in A20 expression (Fig. 1a). Also, we used L929 cells (fibroblast cell line from mouse), in which A20 is highly expressed, to confirm the specificity of A20 staining with the si-RNA to knockdown A20. A20 was knocked down in L929 by 60%–70% after si-RNA treatment (Fig. 1a). Next, to study whether A20 was expressed in tumor stroma, we established E.G7 tumor model in mice with subcutaneous injection of 2 × 10^6^ cells and the immunohistochemical staining of A20 was performed. High expression of A20 was found in the tumor sections, especially in tumor stroma, e.g. in cells with a myeloid derived cell-like morphologies, but not in tumor cells (Fig. 1b). After the treatment of mice with intratumoral injection of si-A20, the A20 positive cells in tumor tissues were significantly decreased (Fig. 1b). Due to the specific morphologies of A20-positive cells, we further investigated the cell type of the A20-expressing cells. The results of immunofluorescence staining showed that the A20-positive cells were also Gr1 positive (Fig. 1c), which indicated that cells expressing A20 could be classified into myeloid derived cells. The efficiencies of two sequences of si-RNA targeting A20 in tissues were evaluated by immunoblotting and the interference efficiencies were also tested using L929 cell line *in vitro*. Both experiments showed that treatment of si-A20 and si-A20′ significantly decreased A20 expression in cells and tumor tissues (Fig. 1d–f). Furthermore, the knockdown of si-A20 and si-A20′ in tumor microenvironment both resulted in the inhibition of tumor growth (Fig. 2a,b).

To evaluate how the down-regulation of A20 affected the tumor microenvironment, tumor tissues were subjected to flow cytometry. Significant decrease in the percentages of MDSCs was found in si-A20 and si-A20′ treated mice while compared with that in si-SCR, si-SCR’ and PBS group (Fig. 2c). This might be due to the high expression of A20 in MDSCs as we found in Fig. 1c. To further understand the tumor inhibitory effect mediated by the down-regulation of A20 in tumor microenvironment, we chose si-A20 for further experiment due to its higher interference efficiency. The percentages of MDSCs decreased after si-A20 treatment on both Day 7 and Day 14 (Fig. 2d). However, the population of dendritic cells, F4/80^+^ macrophages and CD11b^−^ granulocytes were not affected by the injection of si-A20 compared with that of control groups (Fig. 2c,d). Also, the reduction of MDSCs in tumors was confirmed by immunofluorescence staining and the positive cells in each high powered field were counted (Fig. 2e). Moreover, tumor regression was also found in both E.G7 and B16 tumor models after si-A20 treatment (Fig. 2f). These data indicated that down-regulation of A20 in tumor site exerted anti-tumor effect in mice.

### Si-A20 treatment results in enhanced T cell response to overcome tumor-induced tolerance

**Figure 3:**
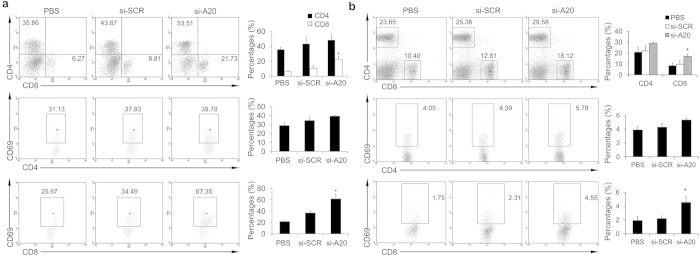
Si-A20 treatment improves T cells activation in tumor-bearing mice. (**a**) Lymphocytes isolated from tumors were subjected to flow cytometry assay (n = 3). Total number of 100000 cells was analyzed. Cells were gated by CD3 lymphocyte region in tumors and cell percentages were illustrated as percentages of the positive cells in gated lymphocytes. For CD69 staining, cells were gated by CD4^+^ or CD8^+^ region. The percentages of positive cells in gated cells were illustrated. Numbers illustrated indicate the percentage of the cells in gated cells. (**b**) Lymphocytes from lymph nodes were evaluated by flow cytometry assay (n = 3). Total number of 30000 cells was analyzed. For CD69 staining, cells were gated by CD4^+^ or CD8^+^ region. The percentages of positive cells in gated cells were illustrated. Numbers illustrated indicate the percentage of the cells in total cells. Data are representative of two independent experiments (n = 3). Data represent means ± SD. *p < 0.05 compared to si-SCR (ANOVA test).

As the treatment of si-A20 showed anti-tumor effect, we further investigated how down-regulation of A20 affected the immune response of the host. Tumors and draining lymph nodes were subjected to flow cytometry after the treatment of mice with si-A20 for two weeks. We found that si-A20 treated group showed a significant increase in CD8^+^ T cells and activated CD69^+^ CD8^+^ T cells in tumor tissues (Fig. 3a). However, the percentages of CD4^+^ T cells were only slightly increased (Fig. 3a). When it comes to the draining lymph nodes, similar results were found with the significant increase in CD8^+^ T cells and activated CD69^+^CD8^+^ T cells, whereas no increase in CD4^+^ T cells and activated CD4^+^CD69^+^ T cells were detected (Fig. 3b). Also, si-A20′ treatment showed the similar effects on T cell response (Supplementary Figure S3).

**Figure 4:**
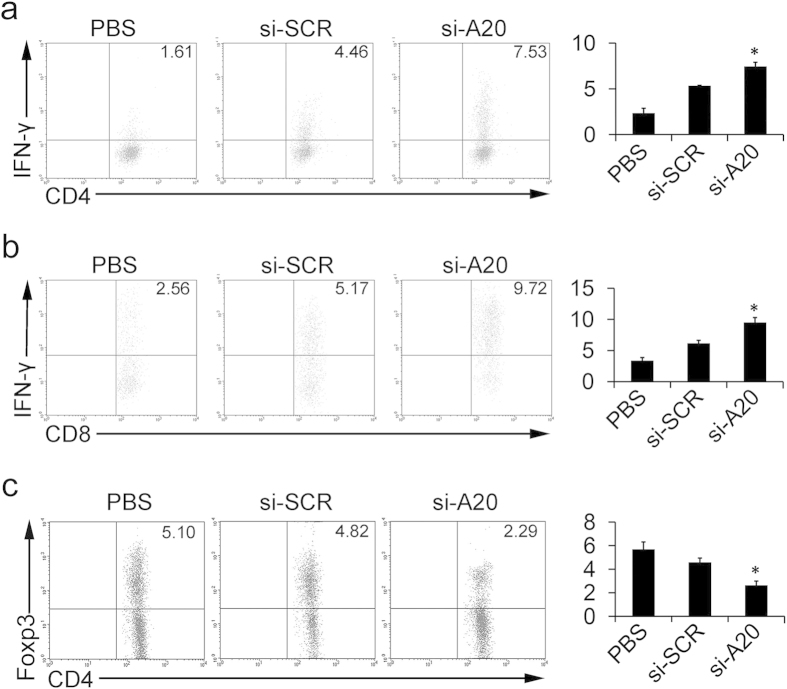
Knockdown of A20 overcomes tumor-induced T-cell tolerance. (**a**,**b**) Analysis of antigen-specific T cell response after the treatment of si-RNA and PBS. T cells isolated from lymph nodes in different groups of mice were subjected to IFN-γ intracellular staining after the re-stimulation with OT-I peptide (**a**) or OT-II peptide (**b**) (n = 3). Total number of 30000 cells was analyzed. Cells were gated by CD4^+^ or CD8^+^ region. Percentages of the positive cells are illustrated. Numbers illustrated indicate the percentage of the cells in total cells. (**c**) Lymphocytes of draining lymph nodes from immunized mice were subjected to Foxp3 intracellular staining (n = 3). Total number of 30000 cells was analyzed. Cells were gated by CD4^+^. Numbers illustrated indicate the percentage of the cells in total cells. Data are representative of two independent experiments (n = 3). Data represent means ± SD. *p < 0.05 compared to si-SCR (ANOVA test).

Moreover, mice treated with si-A20 and si-A20′ showed a significant increase in CD4^+^IFN-γ^+^ and CD8^+^IFN-γ^+^ T cells compared with that in control groups, which indicated that the increase in cytotoxic T lymphocytes (CTL) might also contribute to tumor regression (Fig. 4a,b and Supplementary Figure S4a and b). In addition, we further investigated whether the elimination of MDSCs affected the population of CD4^+^Foxp3^+^ Tregs. The results showed that, si-A20 significantly decreased the percentages of Tregs in tumors, from 4.82% to 2.29% (p < 0.05), which suggested an improved immune response at tumor site after si-A20 treatment (Fig. 4c). Similarly, si-A20′ also decreased the percentages of Tregs in tumor tissues (Supplementary Figure S4c).

**Figure 5:**
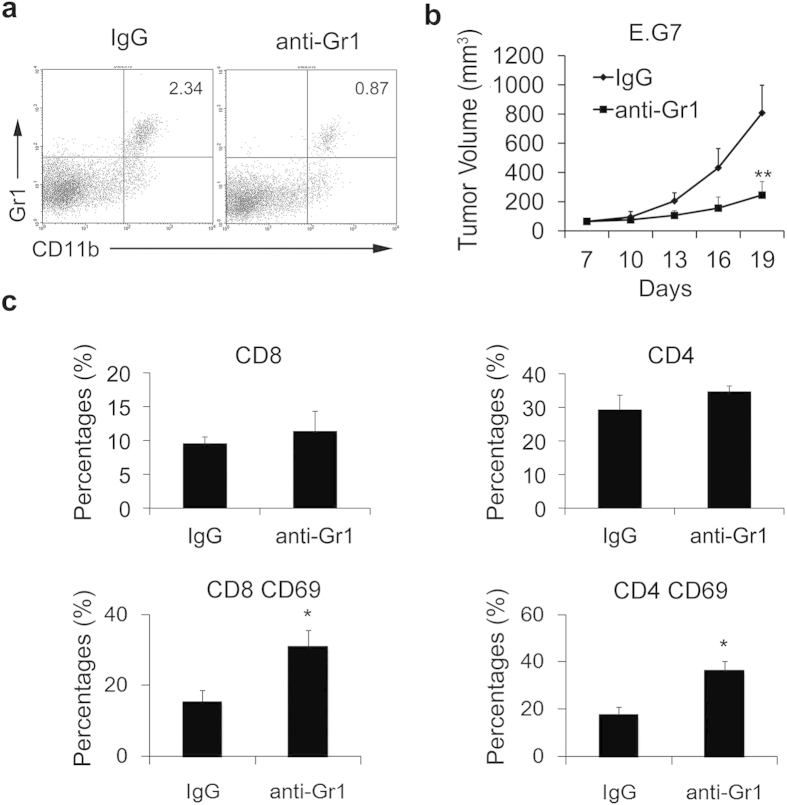
Reduction of MDSC with anti-Gr1 antibody restores T cell activation. (**a**) The efficiency of MDSC elimination by anti-Gr1 antibody. The percentages of Gr1^+^ MDSCs were examined by flow cytometry (*n* = 3). (**b**) Anti-Gr1antibody treatment inhibited E.G7 tumor growth (n = 3). (**c**) The proportion of T cells was measured in tumor tissues in mice treated with anti-IgG and anti-Gr1 antibody. Total number of 100000 cells was analyzed. Cells were gated by CD3 lymphocyte region in tumors. For CD69 staining, cells were gated by CD4^+^ or CD8^+^ region. Numbers illustrated indicate the percentage of the cells in gated cells. Data were representative of three independent experiments (n = 3). Data represent means ± SD. *p < 0.05, **p < 0.01 compared with control group (Student’s *t*-test).

To further support the result that the reduction of MDSCs could enhance T cell response in tumors, we treated tumor-bearing mice with an anti-Gr1 antibody to eliminate MDSCs. The efficiency of elimination was confirmed by flow cytometry (Fig. 5a). Tumor regression was also found in anti-Gr1 antibody-treated mice while compared with the control group (Fig. 5b) and the mice treated with anti-Gr1 antibody also had improved T cell response (Fig. 5c), which was consistent with the changes in tumor volumes and immune responses in mice after the si-A20 treatment. These results suggested that elimination of MDSCs by si-A20 treatment enhanced T cell response, thus, overcoming tumor-induced tolerance and resulting in tumor regression.

### Si-A20 induces the apoptosis of MDSCs *in vitro* and *in vivo*

**Figure 6:**
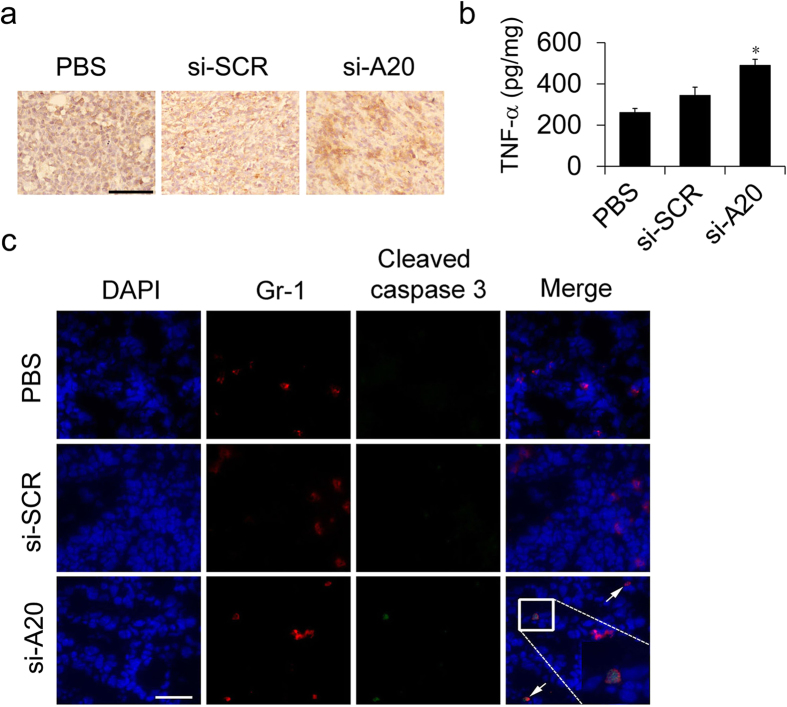
Si-A20 treatment induces the apoptosis of MDSCs *in vivo*. (**a**,**b**) Immunohistochemical and ELISA analysis for the expression of TNF-α in tumor in mice treated with PBS, si-SCR and si-A20 (n = 3). Scale bar 100 μm. (**c**) The expression of cleaved caspase-3 in tumor sections in si-A20 treated mice. Tissues were stained with DAPI (blue), antibodies to Gr1 (Alexa-595) and cleaved caspase-3 (FITC) and analyzed by confocal microscopy, Two independent experiments were performed (*n* = 5). Scale bar 50 μm. Data were representative of two independent experiments. Data represent means ± SD. *p < 0.05 compared to si-SCR (ANOVA test).

Next, we investigated the mechanisms by which si-A20 decreased the number of MDSCs at tumor site. As A20 was reported to inhibit TNF-induced apoptosis in several cell types, we hypothesized that the knockdown of A20 might induce TNF-induced apoptosis in MDSCs. First, we determined the expression of TNF-α at tumor site. The results of immunohistochemical staining showed that TNF-α was significantly highly expressed in the tumors of si-A20 treated mice, whereas, the level of TNF-α was relatively low in the tumors of mice from si-SCR and control group (Fig. 6a,b). Next, we investigated whether the knockdown of A20 could specifically induce cell apoptosis in MDSCs in the presence of TNF-α. The immunofluorescence staining of Gr1 and cleaved caspase-3 in the tumor section was performed. In the si-A20 treatment group, Gr1^+^Caspase-3^+^cells were detected in tumor tissues, whereas, in si-SCR and PBS group, no Gr1^+^ cells were stained as Caspase3-positive (Fig. 6c). Thus, these results suggested that si-A20 induced the apoptosis of MDSCs *in vivo*.

**Figure 7:**
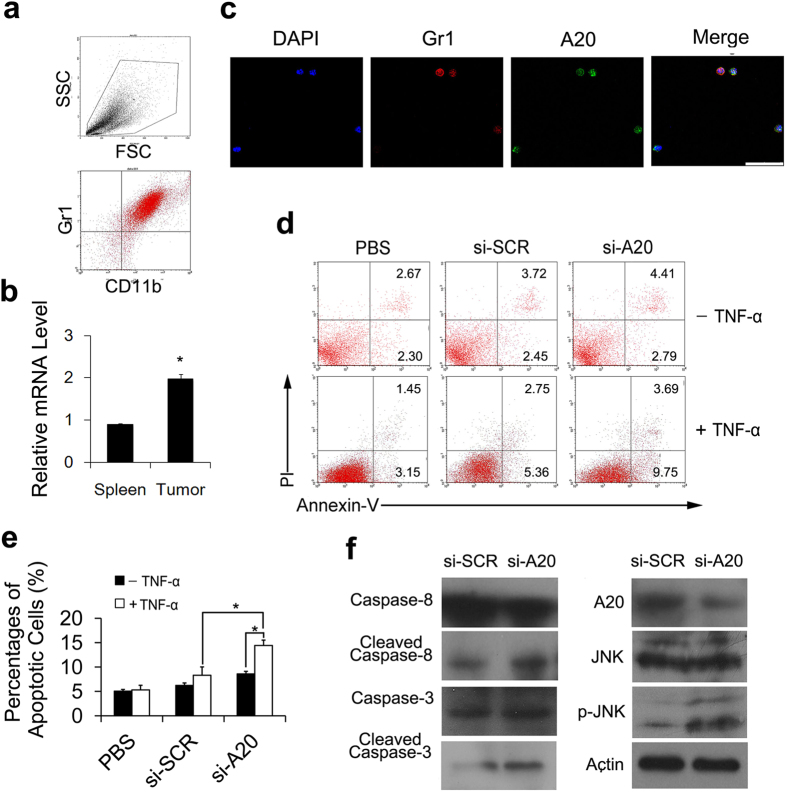
Knockdown of A20 induces the apoptosis of MDSCs through JNK pathway. (**a**) Isolation of MDSCs from tumor tissue was confirmed by flow cytometry. Total number of 30000 cells was analyzed. Numbers illustrated indicate the percentage of the cells in total cells (n = 5). (**b**) mRNA levels of A20 from the isolated MDSCs from spleen and E.G7 tumor were measured (n = 3). (**c**) Fluorescence immunostaining of Gr1 and A20 positive cells in MDSCs isolated from tumor tissue. Scale bar 50 μm. (**d**) Si-A20 treatment induced the apoptosis of MDSC *in vitro*. Gr1^+^CD11b^+^ cells were isolated from tumors in E.G7 tumor-bearing mice and transfected with si-RNA. Apoptosis of MDSCs were confirmed by flow cytometry. Total number of 30000 cells was analyzed. Numbers illustrated indicate the percentage of the cells in total cells. MDSCs were cultured with a mixed medium (TCCM: complete RPMI 1640 = 1:1) with GM-CSF (10 ng/ml). Percentages of the cells in the regions were illustrated. (**e**) Statistic analysis of (**d**). (**f**) Si-A20 induced the apoptosis of MDSCs through JNK pathway. p-JNK, activated caspase-3 and activated caspase-8 were analyzed by western blotting after treatment of MDSCs with si-A20 and TNF-α. Data were representative of two independent experiments. Data represent means ± SD. *p < 0.05 (ANOVA test, Student’s *t*-test).

To find more evidence for the induction of MDSCs apoptosis by si-A20, we tested whether si-A20 could induce MDSCs apoptosis in the presence of TNF-α *in vitro*. The Gr1^+^CD11b^+^ cells were isolated from E.G7-bearing mice using a magnetic bead separation technique and the purity of Gr1^+^CD11b^+^ MDSCs was above 93% (Fig. 7a). Interestingly, we found that the mRNA level of A20 was significantly higher in MDSCs from tumor tissue than that in MDSCs from spleen of tumor-free mice (Fig. 7b). In accordance with Fig. 1c, almost all isolated MDSCs (Gr1 positive) are stained as A20 positive cells with immunofluorescence staining (Fig. 7c). The isolated MDSCs also showed a significant increase in apoptotic cells after being treated with si-A20 and TNF-α *in vitro* while compared with the controls (Fig. 7d,e). Thus, we suggested that si-A20, in the presence of TNF-α, induced the apoptosis of MDSCs both *in vitro* and *in vivo*.

### Si-A20 induces the apoptosis of MDSCs through JNK pathway

To further understand the mechanism underlying how si-A20 induced the apoptosis of MDSCs, we investigated the molecular pathways. After treating isolated MDSCs with si-A20 and TNF-α, cells were subjected to immunoblotting assay. The results revealed a significant increase of p-JNK after si-A20 treatment and activated caspase-3 and caspase-8 were also increased in si-A20 group compared with control groups (Fig. 7f). Therefore, we suggested that the JNK pathway was activated in the induction of MDSCs apoptosis by si-A20.

## Discussion

Several observations have been made in this study concerning tumor microenvironment, A20, MDSCs and cell apoptosis. Our results revealed a high expression of A20 in the tumor microenvironment. The treatment of si-RNA to knockdown A20 expression in tumors resulted in the reduction of MDSCs, which led to an enhanced T cell response, overcoming the tumor induced tolerance and thus exerting an anti-tumor effect. Moreover, the mechanisms by which down-regulation of A20 inhibited tumor growth was that si-A20 induced the apoptosis of MDSCs in the presence of TNF-α through activating JNK pathway. These suggestions are supported by our findings in the study. Namely, the immunohistochemical staining of tumor sections suggested a high expression of A20 in tumor stroma, especially in the cells with myeloid-derived cell-morphologies. Immunofluorescence staining showed the co-localization of the A20-positive cells and Gr1-positive cells both in tumor tissues. The flow cytometry assay indicated a significant decrease in the percentages of MDSCs in tumors after si-A20 treatment while the percentages of dendritic cells and macrophages were not affected compared with control groups. In the meantime, the increases in the percentages of CD8^+^ T cell, CD8^+^CD69^+^ T cell, CD4^+^IFN-γ^+^ T cell and CD8^+^IFN-γ^+^ T cell were found and the percentages of CD4^+^Foxp3^+^ Tregs were significantly decreased. The mice injected with anti-Gr1 antibody to eliminate MDSCs had similar changes in tumor microenvironment to the mice treated with si-A20 and underwent tumor regression. Furthermore, the TNF-α level was elevated after the injection of si-A20 as detected by qRT-PCR. The co-localization of Gr1 and Caspase-3 in tumor section in si-A20 treated mice suggested the induction of MDSCs apoptosis by si-A20. The *in vitro* treatment of isolated MDSCs with si-A20 and TNF-α also showed an increase in cell apoptosis. Western blotting of isolated MDSCs after si-A20 treatment showed the elevated expression of cleaved Caspase-3 and cleaved Caspase-8, also, p-JNK was significantly increased while compared with the controls. On the basis of our findings mentioned above, we suggested that down-regulation of A20 in tumors could eliminate MDSCs through induction of cell apoptosis via JNK pathway, enhancing T cell response and exerting anti-tumor effect.

A20 is well documented as an NF-κB-responsive gene which plays a crucial role in the negative feedback regulation of NF-κB^27^,^28^,^29^. A20 is also recognized as a strong anti-apoptotic factor, because A20-deficient mice(A20^−/−^) were highly susceptible to low doses of TNF and die shortly after birth due to the severe inflammation and tissue damage in multiple organs^21^,^30^. Moreover, the anti-apoptotic effect of A20 seems to be dominant to the cell death sensitizing effect of NF-κB inhibition, as manyA20^−/−^ cell types are highly sensitive to TNF-induced apoptosis, while cells with A20 over- expressed are found to be more resistant to apoptotic cell death induced by TNF/Cycloheximide (CHX). Previous studies showed that knockdown of A20 by specific si-RNA elicited the persistent JNK activation after TNF treatment, which indicated that A20 might play a role in regulating JNK pathway^31^,^32^. Persistent activation of JNK contributed to TNF-induced cell death^33^,^34^. In addition, recent studies also showed that A20 blocked TNF-induced apoptosis through the suppression of JNK by variety of mechanisms^31^,^32^,^35^. In the present study, we focused on the A20 expression in the cells in tumor microenvironment and it is interesting to find that A20 was highly expressed in tumor stroma, in MDSCs. For the first time, we looked into potential role of A20 in tumor growth and knocked down the expression of A20 in tumors to investigate the possible anti-tumor effect. The results showed that down-regulation of A20 inhibited tumor growth and induced apoptosis of MDSCs. The molecular mechanism that induced the apoptosis of MDSCs was also studied. After treating cells with si-A20 and TNF-α, p-JNK increased and the levels of cleaved Caspase-3 and Caspase-8 were also elevated. These results indicated that knockdown of A20 induced Caspase-dependent apoptosis of MDSCs.

MDSCs are important components of tumor microenvironment which expand in cancer bearing hosts and contribute to tumor immune evasion. The important roles of MDSCs in the regulation of tumor development and whose ability to suppress T cell response make them potential targets in cancer therapy^36^,^37^. Several drugs which can reduce or inactivate MDSCs were reported such as gemcitabine^38^, sunitinib^39^ and retinoic acid^40^. However, most of these drugs are chemotherapeutic agents and might cause side effects. In the present study, we found that knockdown of A20 in tumor microenvironment induced the apoptosis of MDSCs, which suggests a new strategy to reduce MDSCs. Due to the phagocytotic function of MDSCs^36^, lipoplexes are more likely to be uptaken by MDCSs rather than other stromal cells or tumor cells. The treatment of mice with si-A20 substantially reduced both the proportion and the absolute number of MDSCs at tumor sites. Also, high levels of TNF-α were detected after si-A20 injection, which is one of the major proinflammatory cytokines and can induce apoptosis in many A20-defect cells. Thus, we deduced that si-A20 decreased the number of MDSCs by inducing cell apoptosis in the presence of TNF-α. Also, we considered that another possible reason for the decreased number of MDSCs could possibly be the induction of MDSC differentiation by si-A20. Previous reports showed that ATRA could induce the differentiation of MDSCs into DCs, macrophages, and granulocytes^40^. We have evaluated the effect of si-A20 on the differentiation of MDSCs *in vivo* by flow cytometry on day 7 and day 14 after the treatment. However, results showed that the numbers of DCs, macrophages did not increase after si-A20 treatment while compared with control groups.

One of the characteristics of MDSCs is the ability to suppress T cell responses. A number of studies have shown that elimination of myeloid suppressor cells could overcome tumor-induced immunosuppression and augment immunotherapy^13^,^40^,^41^. In this study, we found that the proportions of CD8^+^ cells were increased both in tumor tissues and in draining lymph nodes after si-A20 treatment compared with that in control groups. These data were also consistent with the results of depleting MDSCs with anti-Gr1 antibody in tumor-bearing mice. Interestingly, si-A20 treatment only increased the CD8^+^CD69^+^ population compared with anti-Gr1 antibody treatment. One possible reason might be that si-A20 treatment can only lead to cell apoptosis in the specific group of MDSCs which suppress the activity of CD8^+^ T cells. Another possible reason might be that specific deletion of A20 in T cells could only trigger the activity of CD8^+^ T cells^42^. IFN-γ production by CD8^+^ and CD4^+^ T cells was also significantly increased in the si-A20 treated group, while the number of Tregs was reduced compared with the controls. In conclusion, si-A20 treatment could reduce MDSCs in tumor tissues and considerably improve anti-tumor immune response.

In summary, here we have provided insights into the role of A20 in regulating the apoptosis of MDSCs in tumor microenvironment. The knockdown of A20 in tumor tissue induced cell apoptosis in MDSCs via the activation of JNK pathway, thus, improving anti-tumor immune response and exerting anti-tumor effect. However, tumor microenvironment is a complex one. In this study we demonstrated that knockdown of A20 in tumor site using si-RNA can inhibit tumor growth at least through inducing the apoptosis of MDSCs. This may be helpful for seeking a new strategy in anticancer therapy, making A20 inhibitor a potential *in situ* cancer vaccine in the future.

## Materials and Methods

### Cell lines

E.G7, CT26, B16-F10, 4T1 and L929 cells were obtained from American Type Culture Collection (ATCC). E.G7, 4T1, L929 and CT26 cells were grown in RPMI 1640 media plus 10% fetal bovine serum (FBS) and B16-F10 cells were grown in Dulbecco’s Modified Eagle’s Medium plus 10% FBS. To generate tumor cell conditioned medium (TCCM), E.G7 cells were grown in the medium with 3% FBS and without G418 for 48 h^43^. RPMI 1640 and FBS were purchased from Gibco; murine granulocyte-macrophage colony stimulating factor (GM-CSF) and antibiotic were purchased from Sigma.

### Animal models and drug administration

Female C57BL/6 (B6) mice (6–8 weeks) were purchased from HFK Bioscience (Beijing, china). To establish tumor model, mice were injected subcutaneously (s.c.) with 2 × 10^6^ E.G7 cells or 1 × 10^6^ B16-F10 cells. After 7 days, mice with tumors around 60 mm^3^ were selected and randomly divided into various groups. Mice were treated with intratumoral injection of si-A20 and si-SCR at a dose of 10 μg/mice twice a week for three weeks. Controls were treated with sterile PBS. For MDSC depletion with Gr1 antibody, 7 days after E.G7 tumor inoculation, mice were treated intraperitoneally (i.p.) with either anti-Gr1 or isotype control antibody (BD Biosciences) at a concentration of 150 μg/mouse in 200  μl PBS once a week. Tumor size was measured every two days and tumor volume was calculated using the following formula: tumor volume = length × width^2^ × 0.52. For the flow cytometry assay, confocal microscopy and immunohistochemical staining, mice were treated as mentioned above for one or two weeks.

### Isolation of MDSCs from primary tumor

Tumors were cut into small pieces and incubated at 37 °C for 1 hour in 20 ml of RPMI (serum free) containing 1 mg/ml collagenase I (280 U/mg; Gibco) and 2 μl of DNase (2 mg/ml; Sigma). Then the tissue mixtures were passed through a 70-μM filter. Next, the cell suspension was centrifuged at 300 g for 10 minutes. The collected cells were passed through a 70-μM filter again, and centrifuged on Histopaque®-1077 (Sigma) at 400 g for 30 minutes. Live cells were collected from the interface and washed with RPMI (serum free). Gr1 positive cells were isolated from the cell mixture by Miltenyi magnetic bead purification with anti-Gr1 mAbs (RB6-8C5) and LS MACS columns according to manufacturer’s protocol. The isolated cells were analyzed by flow cytometry and the Gr1 positive cells were more than 93%.

### Antibodies, flow cytometry

FITC anti-mouse CD8a, PE or APC anti-mouse CD4, FITC or Percp-Cy 5.5 anti-mouse CD11b, PE anti-mouse CD11c, PE or APC anti-mouse CD69, PE anti-mouse F4/80, PE anti-mouse Foxp3, FITC or PE anti-mouse IFN-γ, FITC or PE anti-mouse Ly-G and Ly-6C, anti-mouse Gr1, and isotype-matched mAbs were purchased from BD Biosciences; anti-JNK antibody and anti-p-JNK antibody were purchased from Cell Signaling Tecnology. Anti-mouse A20 antibody was purchased from Abcam; Alexa Fluor 488-conjugated donkey-anti-rabbit secondary antibody was purchased from Invitrogen. Fixation/Permeabilization Kit was purchased from eBioscience. OT-I peptide and OT-II peptide were purchased from Invivogen. For cell surface staining, cells were directly stained with either IgG control or fluorescence-conjugated antibodies; for intracellular cytokine staining, cells were permeabilized and fixed after surface staining, and stained with fluorescence-conjugated antibodies or IgG controls. Lymph nodes used in experiments were near the tumors. Analysis was carried out on a FACS calibur flow cytometer (BD Biosciences). CD11b positive cell sorting was performed by Aria I (BD Biosciences). Annexin-V-FLOUS Staining Kit was purchased from Roche and cell apoptosis was determined according to the manufacturer’s protocol.

### Confocal microscopy

Three-micrometer frozen section of the treated mice were prepared, fixed and stained with rat-anti-mouse Gr1 antibody (BD PharMingen, 1:50), rabbit-anti-mouse cleaved-Caspase-3 antibody and rabbit-anti-mouse A20 antibody (Abcam, 1:400). The secondary antibodies were Alexa Fluor 488-conjugated donkey-anti-rabbit antibody (Invitrogen, 1:500) and Alexa Fluor 594-conjugated donkey-anti-rat antibody (Invitrogen, 1:100). Samples were also stained with DAPI (5 μg/ml) for 10 minutes, washed and were visualized by a Leica TCS SP5 confocal microscope. Negative control: tumor sections were stained with PBS and secondary antibodies without primary antibodies.

### Synthesis of si-RNA and lipoplexes preparation

Cationic liposomes were made of 1,2-dioleoyloxy-3-trimethylammonium propane (DOTAP)/dioleoylphosphatidylethanolamine (DOPE) and prepared by conventional evaporation method^44^. The A20 si-RNA sequence targeting mouse A20: si-A20 for5′-CAA AGC ACU UAU UGA CAG A-3′^45^ and si-A20′ for 5′-AAC CAT GCA CCG ATA CAC GCT-3′^46^ was synthesized by RiboBio (Guangzhou, china), and the scrambled sequences (si-SCR) were used as controls for off-target siRNA effects. Liposomes loaded with si-RNA were prepared by simply mixing si-RNA and cationic liposomes in PBS. In brief, we diluted 81 μl of liposome (5 mg/ml) by adding 254 μl sterile PBS and diluted 67.5 μg si-RNA by adding 335 μl sterile PBS. The diluted liposome and si-RNA were mixed and incubated at room temperature for 20 minutes, respectively. The obtained lipoplexes were used in the animal experiments.

### Transfection

L929 cells and isolated MDSCs were transfected with synthetic si-A20 or si-SCR by Gene Silencer (San Diego, CA) according to manufacturer’s instruction. Cells were transfected for 48 h were used for subsequent experiments.

### Western Blot

Isolated MDSCs were plated in 6-well plates and transfected with A20 si-RNA or Scramble si-RNA as described above. 48 h later, cells were stimulated with TNF-α (30 ng/ml) for 1 h. Then the cells were harvested, lysed and immunoblotted with antibodies described above^47^.

### Cytokine ELISA

To measure the levels of TNF-α, equal tumor tissues from each group were dissected and grinded using glass Dounce Homogenizerin with lysis buffer and the supernatants were collected. Then, the levels of TNF-α was measured by ELISA assay (eBioscience) according to the manufacturer’s instruction.

### Immunohistochemistry

Tissues were fixed with 4% paraformaldehyde and embedded in paraffin. Then five-micrometer sections were prepared and deparaffinized before immunohistochemical staining. Endogenous peroxide was blocked using 3% H_2_O_2_ for 10 minutes at room temperature. Heat antigen retrieval was done in citrate buffer (pH 6.0) for 3 minutes in an autoclave. Gr1, A20 or TNF-α staining was performed using rat anti-mouse Gr1 Ab (BD Biosciences, 1:50), mouse anti-mouse A20 Ab (Santa 1:100) or rabbit anti-mouse TNF-α Ab (Abcam, 1:100), and a secondary rabbit anti-rat antibody or goat anti-rabbit antibody conjugated to biotin was also used. Then, streptavidin and 3, 3′- diaminobenzidine substrate were added. The Stained slides were examined using an upright microscope (Eclipse 80i, Nikon). Ten randomly selected high power fields/slide. For each site, 3 pathologists performed a blind read of the glass slides.

### Real-Time PCR

Total RNA was prepared using the RNA Simple Total RNA Kit (TIANGEN), and the isolated RNA was reverse transcribed into cDNA using a PrimeScript™ RT reagent Kit with gDNA Eraser (TaKaRa). Primers used were as follows: A20 forward 5′-TTT GCT ACG ACA CTC GGA AC-3′ and reverse 5′-TTC TGA GGA TGT TGC TGA GG-3′; GAPDH forward 5′-CCC AGA AGA CTG TGG ATG G-3′ and reverse 5′-CAC ATT GGG GGT AGG AAC AC-3′. Real-time PCR was performed with Sso Advanced™ SYBR Green Supermix (Bio-Rad) using a Two-step PCR reaction procedure. mRNA levels were measured using a CFX96 Real-Time Systerm (Bio-Rad). Expression of the A20 was normalized to the expression of GAPDH.

### Statistical analysis

Statistics analysis was performed using the SPSS software. Statistical comparisons between groups were performed using Student’s *t*-test or ANOVA test. Data were presented as mean ± SD.

### Ethics statement

The methods were carried out in accordance with the approved guidelines. The Animal Care and Use Committee of Sichuan University (Chengdu, Sichuan, China) approved all the animal experiments.

## Additional Information

**How to cite this article**: Shao, B. *et al.* Inhibition of A20 expression in tumor microenvironment exerts anti-tumor effect through inducing myeloid-derived suppressor cells apoptosis. *Sci. Rep.* **5**, 16437; doi: 10.1038/srep16437 (2015).

## Supplementary Material

Supplementary Information
