# Author Correction: Inhibition of A20 expression in tumor microenvironment exerts anti-tumor effect through inducing myeloid-derived suppressor cells apoptosis

**DOI:** 10.1038/s41598-023-31371-8

**Published:** 2023-03-16

**Authors:** Bin Shao, Xiawei Wei, Min Luo, Jiayun Yu, Aiping Tong, Xuelei Ma, Tinghong Ye, Hongxin Deng, Yaxiong Sang, Xiao Liang, Yu Ma, Qinjie Wu, Wei Du, Jing Du, Xiang Gao, Yi Wen, Ping Fu, Huashan Shi, Shuntao Luo, Yuquan Wei

**Affiliations:** 1 Division of Nephrology of Department of Internal Medicine and Lab of Aging Research, State Key Laboratory of Biotherapy & Collaborative Innovation Center of Biotherapy, West China Hospital, Sichuan University, Chengdu, 610041 Sichuan PR China; 2 College of Life Science, Sichuan University, Chengdu, 610041 China

Correction to: *Scientific Reports* 10.1038/srep16437, published online 12 November 2015

This Article contains an error in the top panel in Figure 3B where the si-SCR panel is a duplication of the si-A20 panel.

The corrected Figure 3 and accompanying legend appear below as Figure 1.

**Figure 1:**
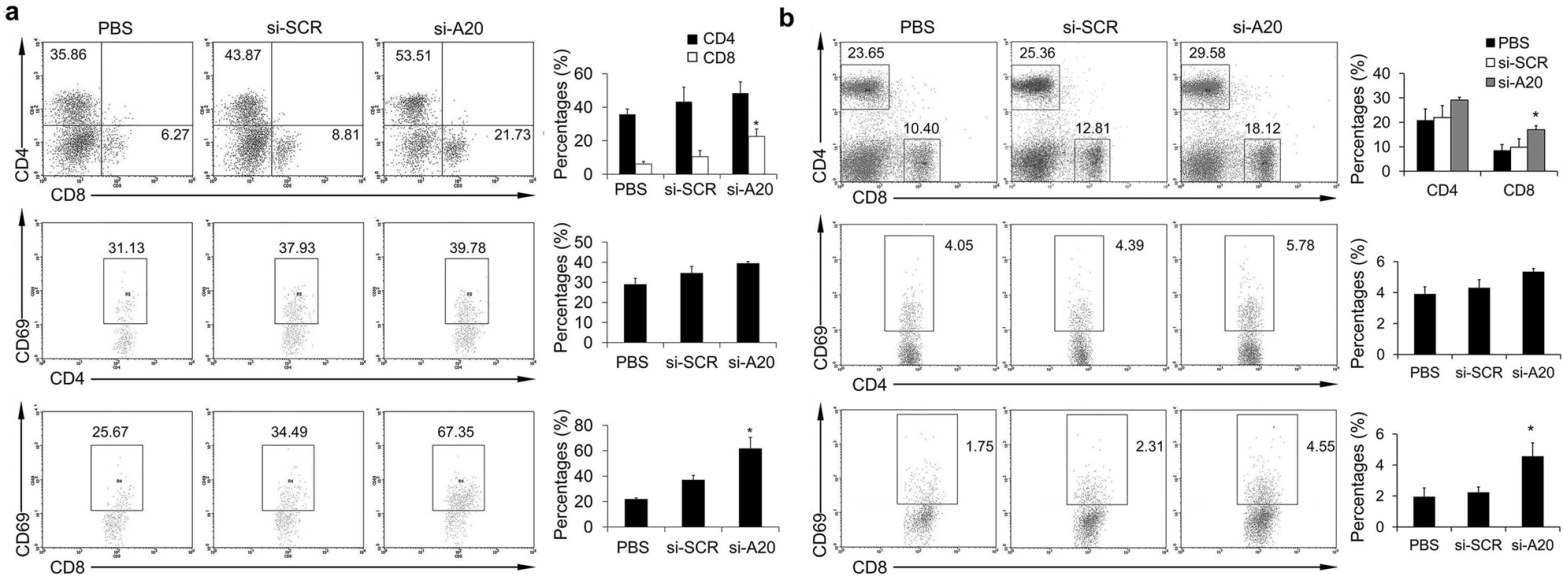
Si-A20 treatment improves T cells activation in tumor-bearing mice. (**a**) Lymphocytes isolated from tumors were subjected to flow cytometry assay (n = 3). Total number of 100000 cells was analyzed. Cells were gated by CD3 lymphocyte region in tumors and cell percentages were illustrated as percentages of the positive cells in gated lymphocytes. For CD69 staining, cells were gated by CD4+ or CD8+ region. The percentages of positive cells in gated cells were illustrated. Numbers illustrated indicate the percentage of the cells in gated cells. (**b**) Lymphocytes from lymph nodes were evaluated by flow cytometry assay (n = 3). Total number of 30000 cells was analyzed. For CD69 staining, cells were gated by CD4+ or CD8+ region. The percentages of positive cells in gated cells were illustrated. Numbers illustrated indicate the percentage of the cells in total cells. Data are representative of two independent experiments (n = 3). Data represent means ± SD. *p < 0.05 compared to si-SCR (ANOVA test).

