# Supplementary material for: Inhibition of A20 expression in tumor microenvironment exerts anti-tumor effect through inducing myeloid-derived suppressor cells apoptosis
Source: Sci Rep. 2015 Nov 12;5:16437. doi: 10.1038/srep16437 (PMC4642332; doi:10.1038/srep16437)
Supplement: Supplementary Information [file srep16437-s1.pdf]

supplementary information

Inhibition of A20 expression in tumor microenvironment exerts anti-tumor effect through inducing myeloid-derived suppressor cells apoptosis

Bin Shao[†1], Xiawei Wei[†1], Min Luo[†], Jiayun Yu[†], Aiping Tong[†], Xuelei Ma[†], Tinghong Ye[†], Hongxin Deng[†], Yaxiong Sang[‡], Xiao Liang[†], Yu Ma[†], Qinjie Wu[†], Wei Du[†], Jing Du[†], Xiang Gao[†], Yi Wen[†], Ping Fu[†], Huashan Shi[†], ShuntaoLuo[†], Yuquan Wei*

[†]State Key Laboratory of Biotherapy and Cancer Center, West China School of Pharmacy, West China Hospital, Sichuan University , and Collaborative Innovation Center for Biotherapy, No. 17, Section 3 Renmin South Road, Chengdu 610041, China.

[‡]College of life science, Sichuan University, Chengdu 610041, China

Running title: A20 inhibition in tumor microenvironment

[1]Bin Shao and Xiawei Wei have contributed equally to this work.

*Correspondence to: Yuquan Wei, Email Addresses: yuquawei@vip.sina.com, yqwei@scu.edu.cn.

Tel.: +86 2885502796; fax: +86 2885502796.

a.

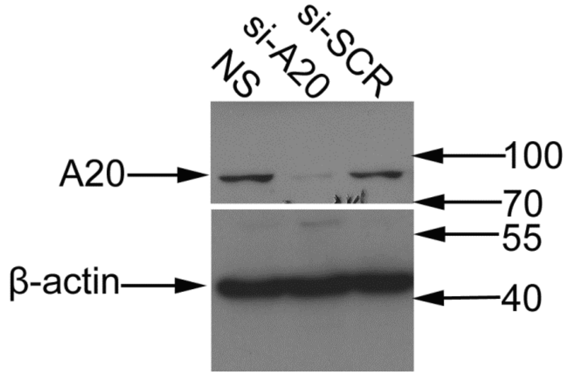

b.

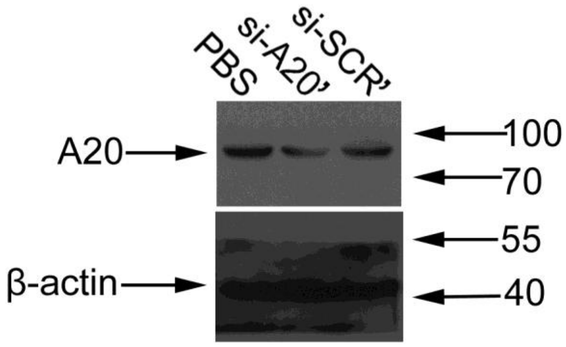

c.

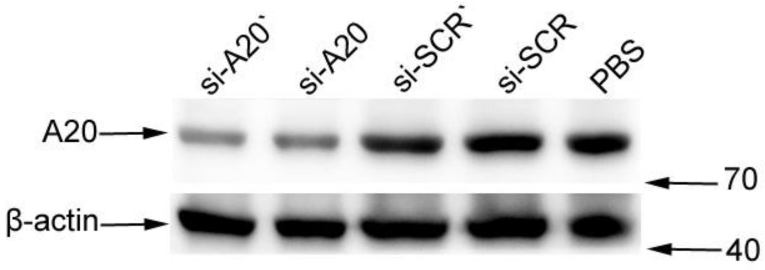

Supplementary Figure S1. The interference efficiency of si-A20 was tested by western blotting in the L929 cells. (a,b) L929 cells were tranfected for 48 hours and then stimulated with TNF-α (30ng/ml) for 1 hour. A20 protein was detected by western blot. β-actin was used as internal control . **(c)** Decrease of A20 expression in tumor tissue after si-A20 treatment. Tumor-bearing mice were treated with si-RNA intratumorally and $CD11b^+$ cells are isolated from pooled tumor tissue suspension (3-5 mice per group). These cropped blots are used in the main figure (Figure 1) and these full-length blots are included in the supplementary figure.

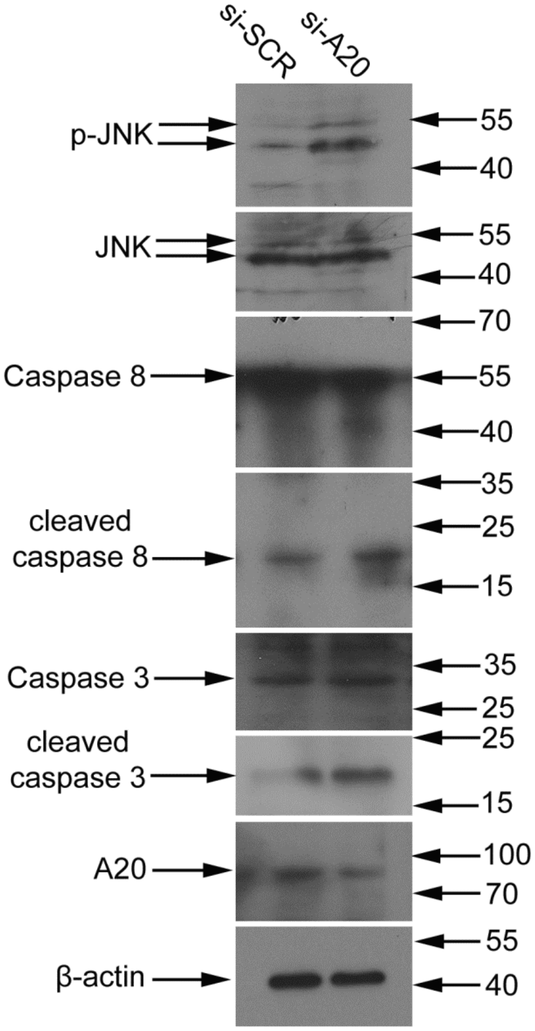

Supplementary Figure S2. Si-A20 induces the apoptosis of MDSCs through JNK pathway. Gr-1$^+$CD11b$^+$ cells were isolated and transfected as mentioned above. p-JNK, activated caspase3 and activated caspase8 were analyzed by western blotting after treatment with si-SCR and si-A20. β-actin was used as internal control . These cropped blots are used in the main figure (Figure 7) and these full-length blots are included in the supplementary figure.

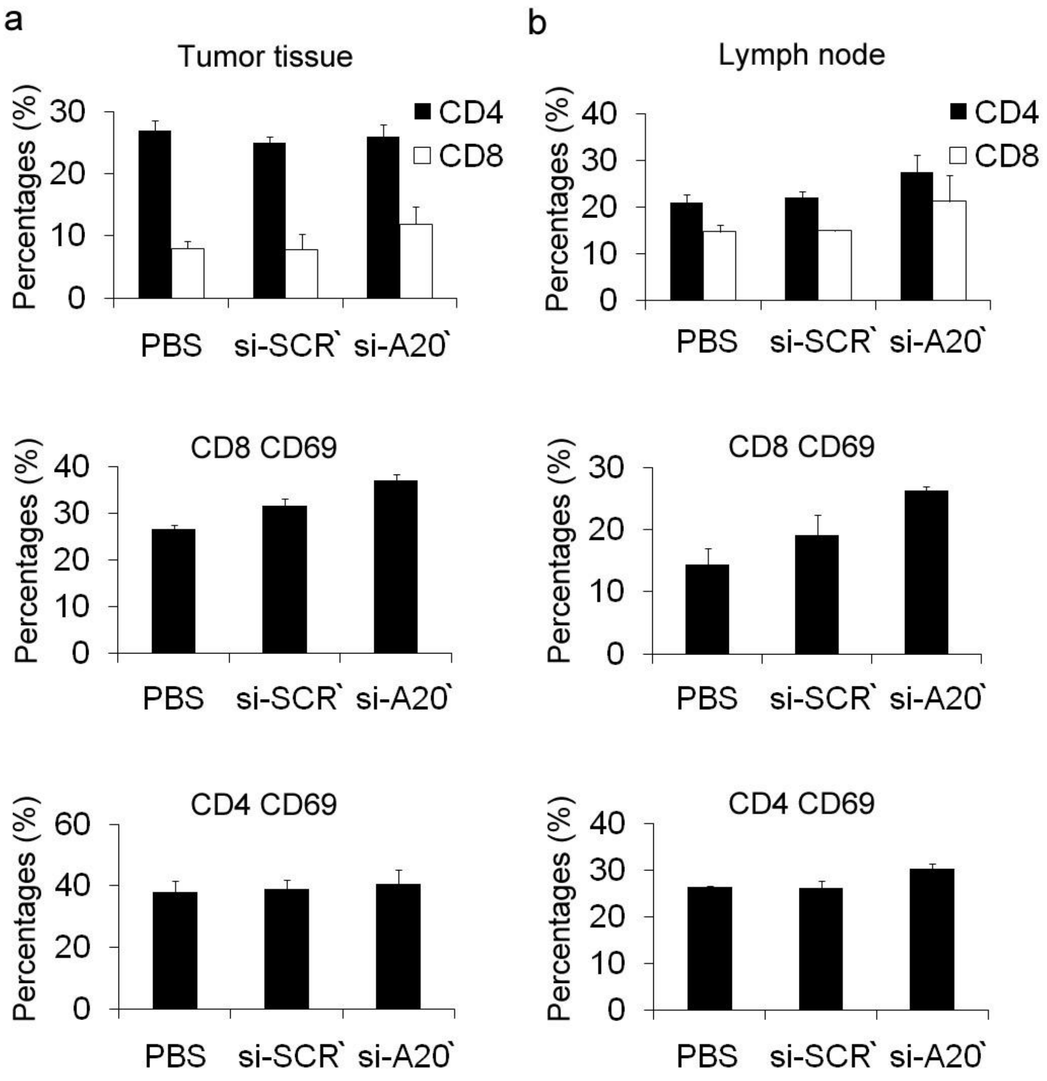

Supplementary Figure S3. Si-A20′ treatment improves T cells activation in tumor-bearing mice. E.G7 tumor model was established and mice were treated with si-A20′ as mentioned above. **(a)** Percentages of T cells in tumor tissue. Total number of 100000 cells were analyzed. Cells were gated by CD3 lymphocyte region in tumors and cell percentages were illustrated as percentages of the positive cells in gated lymphocytes. For CD69 staining, cells were gated by $CD4^+$ or $CD8^+$ region. Numbers illustrated indicate the percentage of the cells in gated cells. n=3 for each group. Two independent experiments were performed. (b) Percentages of T cells in Lymph node. Total number of 30000 cells were subjected to flow cytometry assay. For CD69 staining, cells were gated by $CD4^+$ or $CD8^+$ region. The percentages of positive cells

in gated cells were illustrated. n=3. Two independent experiments were performed. Data represent means ± SD. (ANOVA test).

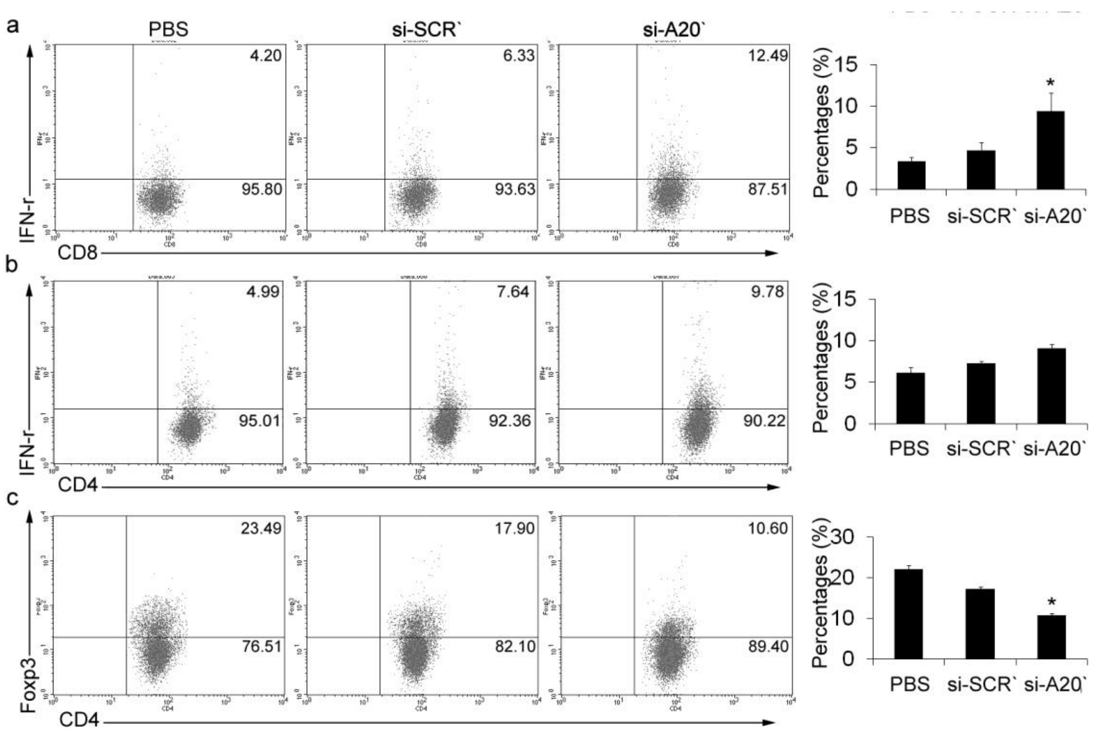

Supplementary Figure S4. Knockdown of A20 using si-A20′ overcomes tumor-induced T-cell tolerance. (a, b) Analysis of antigen-specific T cell response. T cells isolated from lymph nodes were subjected to INF-γ intracellular staining after the re-stimulation with OT-I peptide (a) or OT-II peptide (b). Total number of 30000 cells were analyzed. Cells were gated by $CD4^+$ or $CD8^+$ region. Percentages of the positive cells are illustrated. Numbers illustrated indicate the percentage of the cells in total CD4 or CD8 cells. n=3 for each group. Independent experiments were repeated twice with similar results. Data represent means ± SD. (c) Analysis of Treg cells in draining lymph nodes. Lymphocytes from immunized mice were subjected to Foxp3 intracellular staining. Total number of 30000 cells were analyzed. Cells were gated by CD4 positive region. Numbers illustrated indicate the percentage of the cells in total CD4 cells. n=3 for each group. Two independent experiments were performed. Data represent means ± SD. *$p < 0.05$ (ANOVA test).
